# Preliminary Effectiveness of an Intimate Partner Violence Intervention in Reducing Recidivism Among Criminal Justice-Involved Individuals in Switzerland

**DOI:** 10.1177/08862605251357852

**Published:** 2025-07-30

**Authors:** Madeleine A. Kirschstein, Jérôme Endrass, Astrid Rossegger, Marc Graf, Juliane Gerth

**Affiliations:** 1 Office of Corrections and Rehabilitation, Zurich, Switzerland; 2 University of Basel, Switzerland; 3 University Psychiatric Clinics Basel, Switzerland; 4 University of Konstanz, Germany

**Keywords:** perpetrators of intimate partner violence, batterer intervention program, quasi-experiment, effectiveness evaluation

## Abstract

The recidivism rates of individuals who have perpetrated intimate partner violence (IPV) are high, highlighting the need for effective preventive measures. Although much research has been conducted on this topic abroad, only limited data are available from Switzerland. To address this knowledge gap, the present study evaluated the effectiveness of a Swiss IPV intervention in reducing recidivism among criminal justice-involved individuals. This study is a secondary analysis of data obtained from two previous studies involving samples of adult men who had been police-recorded for IPV. A retrospective quasi-experiment was used to compare the recidivism rates of individuals assigned to the intervention (*n* = 66) to those of individuals not assigned to the program (*n* = 204). Recidivism was defined as any re-offense for IPV recorded by the Cantonal Police of Zurich during a 2-year fixed follow-up period. The IPV recidivism rates were lower in the intervention group (6.06%) than in the control group (22.55%). The Cox proportional hazards regression showed a statistically significant intervention effect (HR = 0.21, *p* = .003, 95% CI [0.07, 0.59]) after adjusting for potential confounders. Given the limitations of this study, these results provide supportive albeit preliminary evidence for the effectiveness of the intervention program. Future research should verify these findings using a methodologically rigorous study design that addresses the limitations of the current study. Refinements of the intervention program and possible new directions for research and practice are discussed.

The lifetime prevalence of intimate partner violence (IPV) is high worldwide. IPV refers to any act of violence perpetrated by a current or former intimate partner that results in physical, sexual, or psychological harm (World Health Organization, 2012). A recent meta-analysis combined the results of 366 population-based surveys in 161 countries using data from 2000 to 2018 (Sardinha et al., 2022). This study found that 27% of women aged 15 to 49 years have experienced physical or sexual violence by an intimate partner in their lifetime, with 13% having been affected in the previous 12 months. According to this study, Switzerland has one of the lowest prevalence rates of IPV in the world (lifetime: 12%, past 12 months: 2%; World Health Organization, 2021). However, the scope of the problem is still considerable. A survey conducted during the coronavirus disease 2019 pandemic that also included psychological violence showed a much higher lifetime prevalence of IPV of about one-third in the Swiss population (42% of women and 24% of men; Bütikofer et al., 2021). Based on police crime statistics, IPV accounted for approximately one-third of police-recorded violence and one-fifth of homicides in Switzerland in 2023 (Federal Statistical Office, 2024b, 2024c), with four-fifths of defendants being men (Federal Statistical Office, 2024d). These data show that, despite a considerable proportion of men being affected by IPV, the majority of violence reported to the authorities, including severe offenses, is perpetrated by men against women.

The high prevalence rates are compounded by the high recidivism rates of individuals who have perpetrated IPV. Meta-analyses based on primary studies with predominantly male samples indicate that recidivism rates for untreated individuals range from 21% based on police records to 35% based on partner reports, while recidivism rates for treated individuals range from 13% based on official records to 26% based on couple reports (Arce et al., 2020; Babcock et al., 2004). In addition, primary studies from Switzerland show that men who underwent a forensic psychiatric evaluation had a recidivism rate of 20% at 8-year follow-up based on judicial records (Seewald et al., 2017), men who were police-recorded for IPV had a recidivism rate of 32% at 5-year follow-up based on police records (Gerth et al., 2017), and men assigned to preventive intervention programs had recidivism rates ranging from 18% at 5-year follow-up based on judicial records to 20% at 1-year follow-up based on police records (Nigl, 2018; Treuthardt & Kröger, 2020). Given these rates, effective measures to prevent IPV are needed.

Apart from campaigns to raise awareness of IPV and its negative consequences, public education on these issues, and training of professionals, intervention programs for individuals who have perpetrated IPV are a common measure to enhance the prevention of IPV (Council of Europe, 2011). According to surveys from North America, these intervention programs are typically delivered by human service professionals to groups of 8 to 10 participants in the community for a predetermined number of sessions once a week, and individuals are referred to these intervention programs by the judicial system (Cannon et al., 2016; Price & Rosenbaum, 2009). At least a dozen systematic reviews and meta-analyses have summarized the body of research on the effectiveness of interventions for individuals who have perpetrated IPV (Arce et al., 2020; Arias et al., 2013; Babcock et al., 2004; Cheng et al., 2021; Davis & Taylor, 1999; Feder & Wilson, 2005; Feder et al., 2008; Karakurt et al., 2019; Santirso et al., 2020; Smedslund et al., 2011; Travers et al., 2021; Wilson et al., 2021). The results of these research syntheses were mixed: Some studies demonstrated statistically significant small to moderate positive effects (Arce et al., 2020; Babcock et al., 2004; Cheng et al., 2021; Davis & Taylor, 1999; Karakurt et al., 2019), while the findings from other studies were unclear, showing either statistically nonsignificant or inconsistent effects (Arias et al., 2013; Feder & Wilson, 2005; Feder et al., 2008; Smedslund et al., 2011; Wilson et al., 2021). In addition, some of the primary studies even produced negative effects, suggesting that the delivery of intervention programs was associated with increased IPV recidivism rates (e.g., Harrell, 1991; Lin et al., 2009). Despite this inconclusive evidence base, these findings do not suggest that IPV interventions are ineffective, as null effects indicating no difference in recidivism rates between study groups were just as likely as sizable positive effects indicating meaningful reductions in the recidivism rate of the intervention group compared to the control group (Arias et al., 2013; Wilson et al., 2021). Rather, these findings imply that there is potential for improvement. Recent research points to possible new directions for refining current interventions.

Two common theoretical approaches to IPV interventions are the Duluth model and cognitive-behavioral therapy (Babcock et al., 2004; Price & Rosenbaum, 2009). While the first approach postulates that men who perpetrate IPV are socialized in a culture that values power and establishes relationships of dominance and submission, and thus men use violence to exert control over women (Pence & Paymar, 1993), the second approach focuses on the relationships between cognitions, emotions, and behavior, and assumes that IPV is learned and results at least in part from distorted thinking patterns (Babcock et al., 2004, 2016). While some meta-analyses have found no evidence that one approach is superior to the other (Arias et al., 2013; Babcock et al., 2004; Davis & Taylor, 1999), other meta-analyses have found mixed effects for the Duluth model and consistent positive effects for cognitive-behavioral therapy (Arce et al., 2020; Karakurt et al., 2019). Further evidence of the superiority of cognitive-behavioral therapy is provided by a meta-analysis showing that interventions that fully adhered to the risk-need-responsivity (RNR) principles (and thus integrate cognitive-behavioral techniques) were more effective in reducing IPV than programs with partial or no adherence (Travers et al., 2021).

The RNR principles specify that the intensity of interventions should correspond to an individual’s level of risk, that programs should positively affect dynamic risk factors for recidivism (and other important needs), that services should integrate cognitive-behavioral and social learning techniques, and that care should match the relevant characteristics of individuals (Andrews & Bonta, 1990; Bonta & Andrews, 2023). Although the Duluth model may be clinically relevant in cases where maladaptive relationship beliefs are related to IPV at the individual level, as a whole it does not align well with these principles. Past researchers have criticized the Duluth model for not being empirically supported, only addressing pro-violent attitudes and not considering other relevant risk factors for IPV, and not being sensitive to diverse populations such as women or same-sex partners who perpetrate violence (Bohall et al., 2016). In comparison, the RNR principles are evidence-based, emphasize the importance of addressing multiple risk factors for recidivism, and account for the heterogeneity of the criminal justice population (Andrews & Bonta, 1990; Bonta & Andrews, 2023).

Findings from recent research overall support the empirical validity of the RNR model and may inform the optimization of current interventions. These studies have identified risk factors that may be particularly relevant for reducing IPV recidivism, as well as specific responsivity considerations for this target population. In addition to risk factors for criminal recidivism in general–such as pro-violent attitudes, the antisocial personality pattern, and substance use–IPV interventions should also address executive functioning, childhood trauma, and stressful life events (Lila & Gilchrist, 2023; Lila et al., 2019). Moreover, IPV interventions should be responsive to participants’ gender (Mackay et al., 2018), sexual orientation (Cannon, 2015; Subirana-Malaret et al., 2019), and racial or ethnic identity (Emezue et al., 2021; Turhan, 2020), and attend to specific subgroups, including individuals who are generally violent, perpetrate certain types of IPV, or use substances (Expósito-Álvarez et al., 2023; Lila et al., 2019; Oğuztüzün et al., 2023). Since program dropout is a strong predictor of IPV recidivism, IPV interventions should also be matched to participants’ motivation for change (Lila et al., 2019). These recommendations are supported by meta-analyses showing that IPV interventions addressing substance use and trauma or incorporating motivational approaches achieve better outcomes than programs that do not (Karakurt et al., 2019; Santirso et al., 2020). However, a recent umbrella review summarized the results of meta-analyses examining whether adherence of services for criminal justice-involved individuals to RNR principles is associated with lower recidivism rates (Fazel et al., 2024). Although the findings provide some support for the RNR model, the review also indicates that the overall quality of the evidence is low and prone to bias, highlighting the need for more methodologically rigorous studies.

Despite much prior investigation into the effectiveness of IPV interventions, none of the primary studies included in the systematic reviews and meta-analyses reported data from Switzerland (see Cheng et al., 2021; Feder & Wilson, 2005; Feder et al., 2008; Karakurt et al., 2019; Santirso et al., 2020; Smedslund et al., 2011; Travers et al., 2021; Wilson et al., 2021). To our knowledge, only two effectiveness evaluations have been conducted in Switzerland to date, with only one having been published in a peer-reviewed journal (Nigl, 2018; Treuthardt & Kröger, 2020). The first study (*N* = 246) investigated men who had perpetrated domestic violence and revealed statistically significantly fewer police records for domestic violence between completers and dropouts at 1-year follow-up (12% vs. 24%). In contrast, the second study (*N* = 192) found only marginally significantly fewer reconvictions for IPV between study groups at 5-year follow-up (14% vs. 25%). Given the limited research on this topic, further studies exploring the effectiveness of these intervention programs in Switzerland are needed.

## The Present Study

This study evaluates the effectiveness of the intervention program “Partnership Without Violence” [Lernprogramm “Partnerschaft ohne Gewalt”] administered by the Probation and Corrections Services of the Office of Corrections and Rehabilitation of the Canton of Zurich. The intervention targets adult men and women who have perpetrated IPV and is similar to the prototypical “batterer intervention programs” in North America (cf., Cannon et al., 2016; Price & Rosenbaum, 2009). Specifically, the program uses cognitive-behavioral therapy techniques, is manualized, and consists of 16 weekly sessions of 2.5 hr each and three follow-up sessions at 3-month intervals, delivered individually or in groups by specialized social workers or psychologists in the community (Regli, 2023). In addition, the intervention program adheres to the RNR principles and applies motivational interviewing to prevent IPV recidivism and promote change toward non-violent behavior (Regli, 2023). The coverage of programs such as this one has expanded in Switzerland in recent years, with at least three-quarters of Swiss cantons now offering this type of intervention (Der Bundesrat, 2021; Treuthardt, 2017).

A retrospective quasi-experiment was used to conduct two group comparisons: (a) Individuals who were assigned to the intervention were compared with those not assigned to the program, and (b) individuals who completed a clinically meaningful number of intervention sessions were compared with those not assigned to the program. We hypothesized that the IPV recidivism rates would be lower in the intervention assignment and completion group than in the control group. This study is based in part on data gathered in an earlier study by Treuthardt and Kröger (2020), which evaluated the effectiveness of this intervention program, but had several limitations with regard to the study design and data analysis. Specifically, the previous study compared only program completers to dropouts, lacking a control group of individuals not assigned to the program; used judicial records as an outcome measure rather than police records; and analyzed the data using bivariate methods, not accounting for the time-to-event data. The present study addresses these limitations and reports timelier data from 2011 to 2016 (and not from 2005 to 2016). In addition, this study may offer a more accurate estimate of the intervention effect than the previous study because the study groups and the outcome measure chosen are less prone to selection and reporting bias (Sterne et al., 2016; Wilson et al., 2021).

## Method

### Reporting Standards

We used the Journal Article Reporting Standards for Quantitative Research (Appelbaum et al., 2018) and the Template for Intervention Description and Replication (TIDieR) checklist and guide (Hoffmann et al., 2014) to improve the quality of reporting. Supplemental Appendix A provides additional information about the quasi-experiment and the intervention.

### Sample

The intervention assignment and completion group were derived from a sample of adult men (*N* = 192) who (a) were recorded for IPV by the Cantonal Police of Zurich between 2002 and 2016, (b) were assigned to the intervention by public prosecutors, courts, or correctional authorities, and (c) were determined eligible to participate in the program by case managers. Conversely, the control group was derived from a sample of adult men (*N* = 588) who (a) were recorded for IPV by the Cantonal Police of Zurich between 2016 and 2017, (b) were administered the Ontario Domestic Assault Risk Assessment (ODARA; Hilton et al., 2004), and (c) were not assigned to the intervention by any of the authorities. The study data were merged from two different research projects. The data for the intervention groups were obtained from the previous study evaluating the effectiveness of this intervention program (Treuthardt & Kröger, 2020), while the data for the control group were obtained from a study determining the predictive validity of the ODARA (Hilton et al., 2004) in Switzerland (Gerth et al., 2023). Hence, all individuals in the control group have perpetrated an index offense involving physical contact or a threat of death with a weapon, as required for scoring this risk assessment instrument (Hilton et al., 2010).

A subset of participants from these initial samples were included in the analysis. For the intervention groups, individuals whose time at risk started before 2011 (*n* = 123) were excluded because records for domestic violence are deleted from the police information system (POLIS) 10 years after the offense (Police Information System Regulation [2005] §18) and their inclusion would have led to an underestimation of recidivism in the intervention group. In addition, two participants in the intervention assignment group were lost to follow-up because they had left Switzerland at an unknown date and therefore had missing outcome data (offenses committed abroad are generally not reported to the Swiss authorities). For the control group, data could not be collected for individuals whose case file was not available at the time of data collection (*n* = 77) because the case fell within the jurisdiction of another canton, proceedings were pending, or the file was inspected by another person. To increase the comparability between study groups, individuals who were recorded by the police in 2017 (*n* = 230), individuals whose cases were processed by the police but not referred to the public prosecutor’s office or whose cases were referred but not proceeded (*n* = 59), and individuals who were sentenced to prison (*n* = 17) were excluded from the control group. For all study groups, individuals who were currently incarcerated (*n* = 1) were excluded from the analysis, as these individuals were not at risk to recidivate. The final sample sizes were *n* = 66 in the intervention assignment group, *n* = 39 in the intervention completion group, and *n* = 204 in the control group. See Supplemental Appendix B for the flow of participants through the quasi-experiment.

### Conditions and Design

In this quasi-experimental design, assignment of participants to conditions was based on selection by the authorities in addition to screening by case managers in the intervention groups (see Supplemental Appendix A for additional information on the intervention procedure). Two intervention effects were estimated in this study: For the effect of assignment to the intervention, all individuals assigned to the intervention were classified as being in the intervention group, regardless of the number of sessions received (intervention assignment group). For the effect of starting and adhering to the intervention, only the subgroup of individuals who completed at least 10 sessions was classified as being in the intervention group (intervention completion group). Both study groups were compared with a control group of individuals not assigned to the program. However, the index offenses may have elicited other responses from the criminal justice system.

### Procedures

For the intervention groups, employees of the Cantonal Police of Zurich collected the recidivism data based on POLIS in January 2021; all other data were collected by employees of the Probation and Corrections Services using their case documentation. For the control group, employees of the Research and Development Division of the Office of Corrections and Rehabilitation of the Canton of Zurich coded all data, including the recidivism data, between October 2020 and May 2021. The recidivism data were based on POLIS. All other data were based on case files from public prosecutors or courts, depending on the authority handling the case. A codebook was developed for data extraction, and research staff were trained in its use prior to data collection. To assess the interrater reliability of the codebook, 3 independent raters coded the same 30 case files. The mean Krippendorff’s alpha-value of the variables in the final codebook that were considered for data analysis was .94 (Mdn = 1.00, *SD* = 0.09), with all variables reaching values of at least .67 and 86.21% reaching values of at least .80.

### Measures

#### Recidivism

We defined recidivism as any re-offense for IPV recorded by the Cantonal Police of Zurich during a 2-year fixed follow-up period. Data were collected on the date, type, and number of re-offenses, and IPV included physical, sexual, and psychological violence perpetrated against a current or former intimate partner.

#### Time at Risk

For the intervention groups, the beginning of the time at risk was the date of assignment to the intervention. The median number of days from the index offense until assignment to the intervention was added to the date of the index offense to obtain a comparable beginning of the time at risk for the control group. The release date from custody was used if individuals were still incarcerated at this time. The end of the time at risk was the date of IPV recidivism or censoring in days. The intervention groups were followed until January 2021 and the control group until June to November 2019. Due to the different follow-up periods of the study groups, the data were right censored if participants did not recidivate during the 2-year follow-up period.

#### Covariates

As the data were obtained from two different research projects, the variables collected for the study groups differ. Variables collected for all study groups include age and nationality of perpetrators and victims, prior convictions for any offense and violent or sexual offenses, and the types of index offenses committed.

### Statistical Analysis

The statistical analysis was conducted in StataIC 16 and involved the following steps: First, descriptive statistics were used to report the participants’ characteristics at baseline. Next, bivariate analyses were performed to test for relationships between baseline characteristics and group membership (the Wilcoxon-Mann-Whitney test for non-normally distributed continuous variables and the chi-square or Fisher’s exact test for categorical variables). Then, two Cox proportional hazards regressions were performed to assess the effectiveness of the intervention in reducing IPV recidivism rates at 2-year follow-up, one for estimating the effect of assignment and one for estimating the effect of starting and adhering to the intervention. We report the results for the univariable model, including group membership only, and a multivariable model including the three main potential confounders.

## Results

### Participant Characteristics

Table 1 presents the participant characteristics at baseline. In terms of the intervention, 24.24% (*n* = 16) of individuals in the intervention group did not start the intervention, 16.67% (*n* = 11) dropped out before session 10, and 59.09% (*n* = 39) partly or fully completed the intervention program. Of those individuals who started the intervention, sessions were often delivered in the group setting (*n* = 30, 60.00%), sometimes in the individual setting (*n* = 11, 22.00%), and sometimes in both settings (*n* = 8, 16.00%). In terms of the criminal justice response to the index offenses, police protective measures were ordered for all individuals in the control group (*n* = 202, 99.02%) and extended for two-fifths of the sample (*n* = 83, 40.69%). These may include domestic exclusion, no-contact, and stay-away orders, which are often issued in conjunction (Protection Against Violence Act [2006] § 3). Furthermore, one-fourth of the control group (*n* = 48, 23.53%) were convicted for the index offense, and one-fifth of the cases were managed by the police in a violence protection procedure (*n* = 39, 19.12%).

**Table 1.** Participant Characteristics at Baseline and Results of the Bivariate Analyses Between Baseline Characteristics and Group Membership.

Baseline Characteristic	Intervention Assignment Group (*n* = 66)	Intervention Completion Group (*n* = 39)^a^	Control Group (*n* = 204)	Comparison IAG vs. CG	Comparison ICG vs. CG
	*n* (%)	*n* (%)	*n* (%)	*z*/*c*^2^	*z*/*c*^2^
Sociodemographics perpetrator					
Age, *M* (*SD*)	37.89 (10.32)	38.65 (10.76)	38.58 (11.68)	0.12	−0.28
Swiss nationality	29 (43.94)	19 (48.72)	82 (40.20)	0.29	0.98
Prior convictions					
Any offenses^b^	36 (54.55)	20 (51.28)	85 (41.67)	0.45	0.03
Violent or sexual offenses^b^	18 (27.27)	9 (23.08)	23 (11.27)	6.36*	2.28
Types of index offenses					
Homicide	—	—	3 (1.47)	n/a	n/a
Assaults^c^	44 (66.67)	24 (61.54)	191 (93.63)	32.13***	33.07***
Threats	43 (65.15)	26 (66.67)	147 (72.06)	1.14	0.46
Deprivation of liberty	4 (6.06)	2 (5.13)	9 (4.41)	n/a	n/a
Coercion	23 (34.85)	12 (30.77)	43 (21.08)	5.12*	1.76
Dangerous acts	—	—	10 (4.90)	n/a	n/a
Insults	1 (1.52)	—	15 (7.35)	n/a	n/a
Sexual violence	2 (3.03)	—	23 (11.27)	n/a	n/a*
Felonies among the index offenses	29 (43.94)	13 (33.33)	103 (50.49)	0.86	3.86*
Sociodemographics victim					
Age, *M* (*SD*)^b^	33.66 (9.92)	34.29 (11.08)	34.93 (11.37)	0.50	0.23
Swiss nationality^b^	35 (53.03)	22 (56.41)	81 (39.71)	4.02*	3.74

*Note.* A dash indicates that none of the participants in the study group met the characteristic. n/a = Fisher’s exact test was used, which has no test statistic. IAG = intervention assignment group; CG = control group; ICG = intervention completion group; *SD* = standard deviation.

a The intervention completion group included individuals who participated in at least 10 sessions of the intervention program.

b The variables on prior convictions (*n* = 33, 12.22%), age of the victim (*n* = 2, 0.74%) and Swiss nationality of the victim (*n* = 1, 0.37%) had missing values.

c Assaults include the index offenses Swiss Criminal Code art. 122, 123, and 126.

* *p* < .05. ***p < .001.

### Bivariate Analyses

Table 1 presents the results of the bivariate analyses between baseline characteristics and group membership. In the bivariate analyses, we found statistically significant differences between the intervention assignment and control group on prior convictions for violent or sexual offenses, the index offenses assaults and coercion, and the nationality of victims. Statistically significant differences between the intervention completion and the control group were found for assaults, sexual violence, and felonies among the index offenses.

The IPV recidivism rates at 2-year follow-up were 6.06% (*n* = 4) in the intervention assignment and 22.55% (*n* = 46) in the control group. The absolute and relative recidivism reduction were 16.49% and 73.13%, respectively. Moreover, 7.69% (*n* = 3) of completers and 3.70% (*n* = 1) of non-completers recidivated during the 2-year fixed follow-up period.

### Survival Analysis

**Figure 1.**
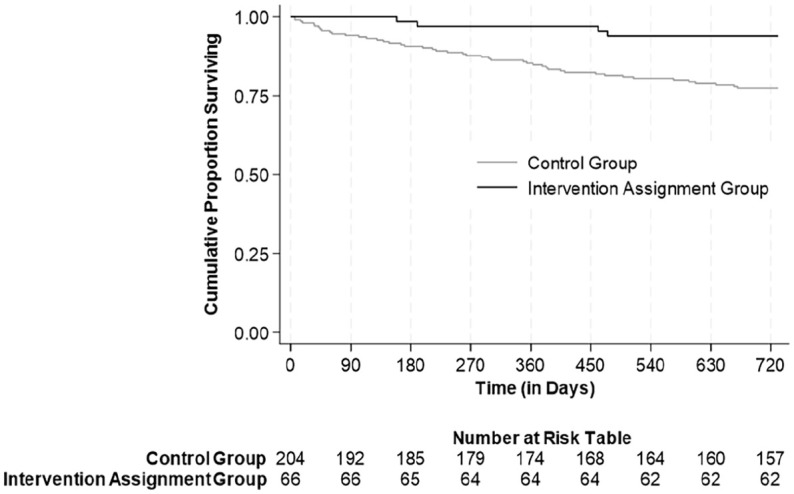
Kaplan-Meier survivor functions for the intervention assignment and control group. *Note.* This figure shows the estimated survival probabilities over time for the study groups. The survivor function is the cumulative proportion of participants not recidivating with intimate partner violence to the beginning of a certain time interval (Tabachnick & Fidell, 2014).

**Figure 2.**
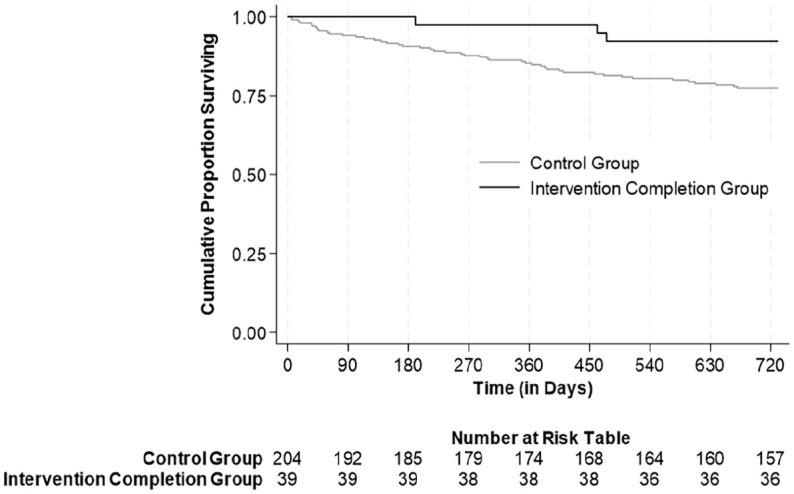
Kaplan-Meier survivor functions for the intervention completion and control group. *Note.* This figure shows the estimated survival probabilities over time for the study groups. The survivor function is the cumulative proportion of participants not recidivating with intimate partner violence to the beginning of a certain time interval (Tabachnick & Fidell, 2014).

For the comparison between the intervention assignment and control group, group membership was statistically significant in the univariable model (χ^2^[1, *N* = 270] = 10.90, *p* = .001) and multivariable model adjusting for potential confounders (χ^2^[4, *N* = 237] = 20.34, *p* < .001). For the comparison between the intervention completion and control group, group membership was also statistically significant in the univariable model (χ^2^[1, *N* = 243] = 5.53, *p* = .019) and multivariable model (χ^2^[4, *N* = 210] = 13.57, *p* = .009). None of the other co-variates were statistically significant in the multivariable models for both group comparisons, although the nationality of perpetrators was marginally significant. Table 2 presents the results of the univariable and multivariable Cox proportional hazards regression analyses for both group comparisons, including hazard ratios (HR), standard errors, *z* values, *p* values, and 95% confidence intervals for each covariate. Compared to being in the control group, being in the intervention assignment and completion group decreased the risk of IPV recidivism by about 79% (HR = 0.21, 95% CI [0.07, 0.59], *p* = .003) and 71% (HR = 0.29 [0.09, 0.93], *p* = .038), respectively. Figures 1 and 2 show the Kaplan-Meier survivor functions.

**Table 2.** Results of the Univariable and Multivariable Cox Proportional Hazards Regression Analyses for Both Group Comparisons.

Variable	HR	SE	*z*	*p*	95% CI
Comparison of intervention assignment vs. control group					
Univariable Cox proportional hazards model					
Group membership	0.24	0.13	−2.71	.007	[0.09, 0.68]
Multivariable Cox proportional hazards model					
Group membership	0.21	0.11	−2.95	.003	[0.07, 0.59]
Perpetrator age^a^	0.49	0.26	−1.35	.178	[0.18, 1.38]
Perpetrator Swiss nationality	0.52	0.18	−1.93	.053	[0.27, 1.01]
Prior convictions for violent or sexual offenses	1.57	0.57	1.24	.215	[0.77, 3.21]
Comparison of intervention completion vs. control group					
Univariable Cox proportional hazards model					
Group membership	0.31	0.18	−1.97	.049	[0.10, 0.99]
Multivariable Cox proportional hazards model					
Group membership	0.29	0.17	−2.07	.038	[0.09, 0.93]
Perpetrator age^a^	0.52	0.27	−1.25	.212	[0.18, 1.46]
Perpetrator Swiss nationality	0.52	0.18	−1.90	.057	[0.27, 1.02]
Prior convictions for violent or sexual offenses	1.48	0.56	1.03	.304	[0.70, 3.10]

*Note.* The number of events was 50 in the first and 49 in the second univariable model. The sample used to estimate the multivariable Cox proportional hazards models for the comparison between the intervention assignment (*n* = 66) and control group (*n* = 171) and for the comparison between the intervention completion (*n* = 39) and control group (*n* = 171) was reduced due to missing values. The number of events was 45 in the first and 44 in the second multivariable model. HR = hazard ratio; CI = confidence interval; SE = standard error.

a Perpetrator age was not normally distributed and thus, log transformed.

We conducted sensitivity analyses by repeating the statistical models with and without missing data to assess the robustness of the study results. In the multivariable models on the multiple imputed data, group membership was only marginally significant for the comparison of the intervention completion and control group, while the nationality of perpetrators reached statistical significance for both group comparisons. Overall, the results were similar across both analyses. See Supplemental Appendix C for the results of the sensitivity analyses and their discussion.

## Discussion

The aim of the present study was to explore the effectiveness of an intervention program for individuals who have perpetrated IPV in Switzerland. To this end, we used a retrospective quasi-experiment to compare (a) individuals assigned to the intervention and determined eligible to participate (intervention assignment group) and (b) individuals who completed a clinically meaningful number of intervention sessions (intervention completion group) with those not assigned to the program (control group). Taken together, the study results provide supporting albeit preliminary evidence for the effectiveness of the intervention program. Our findings support the hypotheses that the IPV recidivism rates at 2-year follow-up were lower in the intervention assignment and completion group than in the control group. The HR were relatively large, indicating that the rate of IPV recidivism decreased by 71% to 79% in the intervention groups compared with the control group. These results are relevant for the economic efficiency of the intervention program. If the costs for IPV possibly prevented by the intervention are compared with the costs of the program, a positive cost-benefit ratio of 1 to 7 is achieved, suggesting that for every Swiss franc invested, seven Swiss francs are returned (see Supplemental Appendix D for the equations of the cost-benefit ratio).

The two intervention effects estimated in this study answer different questions. The comparison between the intervention assignment and control group indicates that *assigning individuals* who were recorded for IPV by the police to the intervention is associated with a lower IPV recidivism rate, regardless of the number of sessions completed. By contrast, the comparison between the intervention completion and control group indicates that *completing a clinically meaningful number of intervention sessions* (>9) is associated with a lower IPV recidivism rate. Both of these intervention effects have to be interpreted with caution. Given the number of events in these study groups, we could only control for the three main potential confounders in the regression analyses.

For the interpretation of these effects, it is important to note that the comparator of the intervention program was not no intervention. The police ordered protective measures, like no-contact orders, against everyone in the control group. Based on relevant data, it can be estimated that about one-fourth of men for whom protective measures are ordered make use of voluntary in-person counseling services for violence prevention, with the proportion being even higher if telephone services are included (Mottl, 2018). In addition, one-fifth of the cases in the control group were managed by the police in a violence protection procedure. Apart from risk assessment, monitoring, and management, this process usually involves the police entering into dialogue with the individual at risk of committing IPV (Greuter, 2018). Given that the control group has also received preventive measures, the results of this study seem to suggest that the intervention program is more effective than lower-threshold interventions.

Two results from this study should be further investigated in future research. Firstly, assignment to the intervention is associated with a lower IPV recidivism rate, although one-fourth of individuals did not start the program. Eligibility of individuals is determined after referral to the intervention and before the start of the program. The screening is based on case files and interviews with assigned individuals, and includes a risk assessment following a structured professional judgment approach, along with personalized feedback on risk factors for recidivism. It is possible that this process already serves as a brief intervention, positively impacting IPV recidivism rates independently of the intervention sessions. As such, individuals who were assigned to the intervention but did not start the program may show lower recidivism rates than those not assigned to the intervention. Second, the IPV recidivism rate of the intervention assignment group, including non-completers is similar to that of the intervention completion group, excluding non-completers. Future research should explore whether this finding is an artifact of the small sample size and the extremely small number of events in the intervention groups, where one re-offense more or less leads to a change in the IPV recidivism rate of several percentage points. An alternative explanation could be that completion of the intervention has no additional effect over assignment to the program.

### Limitations

Two limitations of this study are important to acknowledge, the first of which concerns the comparability of the study groups. The present study evaluated the effectiveness of an intervention program using a retrospective, non-randomized study design, in which receipt of the intervention depended on referral by public prosecutors, judges, or correctional authority staff in addition to the eligibility assessment of case managers. This assignment process may have led to differences between study groups on variables related to IPV recidivism. Although the intervention assignment and completion group only included eligible individuals, the control group included eligible and non-eligible individuals. Given the eligibility criteria, individuals in the intervention groups may have been better integrated into society (due to the language requirement), may have shown fewer mental disorders, may have committed fewer serious violent offenses, and may have committed fewer sexual offenses than the control group.

However, it is reasonable to assume that a considerable proportion of the control group would also have met the eligibility criteria. In light of the high number of police-recorded IPV offenses per year (Federal Statistical Office, 2024c) and the comparatively low number of referrals to the intervention program during the study period, it is likely that public prosecutors, judges, and correctional authority staff did not routinely screen individuals for participation in the intervention but rather ordered attendance of the program sporadically and not systematically. Furthermore, the data indicate that the study groups were comparable for the majority of variables collected. To minimize selection bias, we excluded individuals from the control group who differed from the intervention groups in terms of their stage in the criminal justice system and adjusted for potential confounders in the regression analyses.

The second limitation of this study concerns the different time points of follow-up between the study groups. Due to the small number of individuals referred to the intervention program per year, we had to include cohorts of several years to achieve an adequate sample size for the intervention groups, whereas we included only one cohort for the control group. Therefore, the time points for the 2-year fixed follow-up varied between the intervention groups (2011–2020) and the control group (2016–2019). The number of police-recorded IPV offenses increased by 31.58% from 12,123 in 2011 to 15,951 in 2020 (Federal Statistical Office, 2024c). This increase may be explained at least partly by changes in victim reporting (e.g., due to increased awareness and decreased acceptability of IPV among the general public; Juarros-Basterretxea et al., 2024), in the handling of cases by the authorities (Xie et al., 2012), or in the recording of IPV in POLIS (Federal Statistical Office, 2024a) and thus may have led to a greater underestimation of the IPV recidivism rate in the intervention groups than in the control group. Partly, this increase may also reflect a true increase in the crime rate of IPV and thus may have led to a higher number of police-recorded IPV offenses in the control group.

### Study Implications

Despite these limitations, the results of this study have several implications for research and practice. Research on the effectiveness of IPV interventions outside of North America is limited, and, to our knowledge, only two evaluations have been conducted in Switzerland to date (Nigl, 2018; Treuthardt & Kröger, 2020). The fact that few Swiss intervention programs for individuals who have perpetrated IPV have been assessed for their success in reducing recidivism was recently criticized by a group of experts evaluating the implementation of the provisions of the Istanbul Convention (Group of Experts on Action against Violence against Women and Domestic Violence, 2022). As such, this study makes an important contribution to the literature by extending the current body of evidence on IPV interventions in Switzerland.

The results also show that medium-threshold preventive interventions that integrate empirically supported principles may effectively reduce IPV recidivism and that longer-term treatment is not always indicated. The intervention program evaluated in this study is intermediate between counseling and psychotherapy and constitutes a valid addition to the Swiss service landscape for individuals who have perpetrated IPV (Eidgenössisches Büro für die Gleichstellung von Frau und Mann, 2020). However, given the study limitations, future research using rigorous research methodology is needed to strengthen the evidence for this type of intervention. A recent review identified limitations of previous randomized controlled trials on interventions for individuals who have perpetrated IPV, thereby providing guidance for the design of effectiveness evaluations (Turner et al., 2023). Future research on the intervention program evaluated herein should use a prospective design, include a larger sample, and assess further variables related to recidivism to more comprehensively control for confounding.

Furthermore, development and research on the intervention program should attend to the diversity of the target population. A large proportion of individuals accused of domestic violence in Switzerland are foreign nationals (Federal Statistical Office, 2024d). To overcome language barriers to participation in the intervention, the program has recently been translated into the most commonly spoken languages of the target population. In addition, women were not included in this study because of the extremely low number of referrals to the intervention program within the study period. This is unexpected given that about one-fifth of individuals accused of IPV in Switzerland are women (Federal Statistical Office, 2024d). It is important to ensure that all individuals for whom the intervention is intended have access to the program, regardless of their gender or other characteristics, and that the intervention program is adapted to specific responsivity factors in order to be effective (Bonta & Andrews, 2023).

## Conclusion

Overall, the results of this study suggest that individuals assigned to the intervention and those completing a clinically meaningful number of intervention sessions recidivated at a lower rate than those not assigned to the program. However, this result is preliminary. Future research should address the limitations of this study to provide definitive evidence of the effectiveness of this intervention program.

## Supplemental Material

sj-docx-1-jiv-10.1177_08862605251357852 – Supplemental material for Preliminary Effectiveness of an Intimate Partner Violence Intervention in Reducing Recidivism Among Criminal Justice-Involved Individuals in Switzerland Supplemental material, sj-docx-1-jiv-10.1177_08862605251357852 for Preliminary Effectiveness of an Intimate Partner Violence Intervention in Reducing Recidivism Among Criminal Justice-Involved Individuals in Switzerland by Madeleine A. Kirschstein, Jérôme Endrass, Astrid Rossegger, Marc Graf and Juliane Gerth in Journal of Interpersonal Violence
