# Supplementary material for: Preliminary Effectiveness of an Intimate Partner Violence Intervention in Reducing Recidivism Among Criminal Justice-Involved Individuals in Switzerland
Source: J Interpers Violence. 2025 Jul 30;41(15-16):5675–99. doi: 10.1177/08862605251357852 (PMC13373294; doi:10.1177/08862605251357852)
Supplement: sj-docx-1-jiv-10.1177_08862605251357852 – Supplemental material for Preliminary Effectiveness of an Intimate Partner Violence Intervention in Reducing Recidivism Among Criminal Justice-Involved Individuals in Switzerland [file sj-docx-1-jiv-10.1177_08862605251357852.docx]

**Appendix A**

**Additional Information About the Quasi-Experiment and the Intervention**

The following sections provide additional information about the quasi-experiment and the intervention based on the items of the Journal Article Reporting Standards for Quantitative Research (JARS-Quant; Appelbaum et al., 2018) and the Template for Intervention Description and Replication (TIDieR) checklist and guide (Hoffmann et al., 2014), respectively.

**Quasi-Experiment**

***Method***

**Ethics Approval.** Ethics approval was not obtained for this study because quality assurance projects do not require ethics approval under Swiss law (Schweizerische Ethikkommissionen für die Forschung am Menschen, 2020). This study complied with relevant data protection regulations.

**Masking.** This study was conducted retrospectively and the intention for research was formed after the intervention had been delivered. Therefore, the study participants and all individuals involved in the intervention procedure, including those referring and screening participants for eligibility as well as those delivering the intervention, were unaware that the program was going to be evaluated and that the intervention group was going to be compared with a control group of individuals not assigned to the program. The outcome assessors for the intervention group were aware that they collected recidivism data for individuals who were assigned to the intervention program and the outcome assessors for the control group were aware that they collected recidivism data for individuals who were police-recorded for intimate partner violence (IPV). However, outcome assessors were not aware whether the individuals had received an intervention or not and that the individuals would be included in an effective­ness evaluation, in which they would be compared to another group. Also, objective outcomes such as recidivism are less prone to bias than subjective outcomes (Sterne et al., 2016). By contrast, the researchers analyzing the data and preparing the report were aware of study conditions.

**Data Diagnostics.** One case was excluded from the statistical analysis due to a high number of missing values. Most variables reported in this study had no missing values and except for the variables on prior convictions, variables with missing data had only one or two missing data points scattered throughout cases. Missing values were concentrated in the variables on prior convictions because official criminal records were not available in about one-sixth of case files in the control group. A dummy variable of cases with missing and no missing values on prior convictions was generated to test whether missingness was related to other variables in the data set. Although we found statistically significant relationships between missingness and variables that generally indicate lower severity of the index offense, IPV recidivism was not related to missingness and we therefore concluded that the data were missing at random. Missing values were estimated using multiple imputation.

To improve the modelling, we tested the covariates for normality, linearity, and homoscedasticity. Perpetrator and victim age were not normally distributed and therefore log transformed. The log transformation was the transformation of choice based on skewness and kurtosis tests for normality and on the shape of the distribution and normalized the distributions. Univariate outliers were defined as observations with z scores above 3.29 or below ‑3.29 on one variable, and multivariate outliers were defined as observations with statistically significant χ2 values (p < .001) for Mahalanobis distance (Tabachnick & Fidell, 2014). The transformation reduced the number and deviation of outliers. Only one univariate outlier was detected for victim age and no multivariate outliers were detected. However, several index offenses had 90-10 splits between categories. These variables were not used in the statistical analysis. Based on the bivariate scatterplot, perpetrator and victim age were linearly related. No multicollinearity was found between covariates based on squared multiple correlations of .90 and higher.

**Experimental Manipulations.** Information on the number of professionals delivering the intervention during the study period and the number of program deliveries per professional were not available.

**Units of Delivery and Analysis.** The intervention was delivered in individual, group, or both settings. However, the unit analyzed was the individual. Information on which individuals were in the same group during the delivery of the program was not available.

***Results***

**Sample Size, Power, and Precision.** A post hoc power analysis indicated that given the achieved sample size (*N* = 237), effect size for the intervention (*b_1_* = ‑1.56), significance level (*p* = .05), probability of IPV recidivism (18.99%), and squared multiple correlation of group membership with other covariates (R^2^ = .03), the power of a one-sided test was 100% in the unimputed multivariable model for the comparison of the intervention and control group.

**Statistics and Data Analysis.** We tested the proportionality assumption of the Cox proportional hazards models by inspecting the survival curves, including time‑dependent covariates in the models, and assessing goodness of fit using the Schoenfeld residuals (Tabachnick & Fidell, 2014). These diagnostics indicated that the proportionality assumption was not violated for any of the variables in the regression analyses.

**Intervention**

The information provided in the following sections was derived from a recent publication (Regli, 2023) and personal communication with the head of the department delivering this intervention (J. Regli, personal communication, December 06, 2024), the institutional website (Probation and Corrections Services, 2024a), information materials on the intervention and the training of intervention providers (Probation and Corrections Services, 2020, 2023b, 2024c, 2024b), and the workbooks for participants (Probation and Corrections Services, 2013, 2023a).

***Item 1: Intervention Name***

The name of the intervention program is ‘Partnership Without Violence’ [Lern­programm ‘Partnerschaft ohne Gewalt’].

***Item 2: Rationale, Theory, and Intervention Goal***

The intervention integrates cognitive‑behavioral therapy and motivational interviewing techniques and adheres to the risk-need-responsivity principles. The intervention targets adult men and women who have perpetrated IPV and is intended to reduce IPV recidivism. Several meta-analyses have shown that services that adhere to the risk-need-responsivity principles are effective in reducing the recidivism rates of criminal justice-involved individuals (Andrews et al., 1990; Koehler et al., 2013) and that these findings also apply to specific subtypes of offenders such as individuals who perpetrated IPV (Gutierrez et al., 2017; Travers et al., 2021).

First, the risk principle states that the intensity of services should be adapted to the risk level of individuals (Andrews & Bonta, 1990; Bonta & Andrews, 2023). The intervention is intermediate between counseling and psychotherapy and targets individuals with a moderate risk for IPV recidivism. Second, the need principle states that services should reduce dynamic risk factors and increase protective factors for recidivism (Andrews & Bonta, 1990; Bonta & Andrews, 2023). The intervention aims to promote positive change, particularly with regard to the antisocial personality pattern, pro-violent attitudes, family and intimate relationships, and substance use. Third, the general and specific responsivity principles state that services should integrate cognitive-behavioral and social learning techniques and that care should match the relevant characteristics of individuals (Andrews & Bonta, 1990; Bonta & Andrews, 2023). Using cognitive-behavioral techniques is consistent with the risk-need-responsivity model, as is using motivational interviewing to increase the completion rates of the intervention among individuals with low motivation.

***Item 3: Intervention Materials***

The intervention is delivered according to a manual and the participants receive a workbook. The workbook contains information provided during the intervention and worksheets with exercises to be performed between sessions.

***Item 4: Intervention Procedure***

The intervention procedure involves the following steps: referral, screening, intervention, and follow-up. First, individuals are referred to the intervention program by public prosecutors or courts in the Canton of Zurich or by correctional authorities in Switzerland. These authorities may refer individuals to the intervention at various stages of the criminal justice process­—for example as an alternative measure for pre-trial or preventive detention (Swiss Criminal Procedure Code art. 237) or as a condition for probation (Swiss Criminal Code art. 44) or parole (Swiss Criminal Code art. 87). Individuals who would like to participate at their own request are referred to other programs in the Canton of Zurich that are outside the criminal justice system. At the time the study was conducted, the intervention program was still relatively unknown. This is reflected in the comparatively low number of referrals to the intervention during this period, in contrast to the high number of police-recorded IPV offenses per year (Federal Statistical Office, 2024c). Consequently, it seems that public prosecutors, judges, and correctional authority staff did not routinely screen individuals for participation in the program but rather ordered attendance sporadically and not systematically. Apart from the explicit eligibility criteria for participation in the intervention (see next paragraph), no explicit criteria for the referral of individuals who perpetrated IPV to the program were established by the authorities at the time the study was conducted. However, in the Swiss service landscape, the intervention program is intermediate between counseling and psycho­therapy (Eidgenössisches Büro für die Gleichstellung von Frau und Mann, 2020), making it an appropriate option for individuals whose risk level for recidivism requires higher intensity services than counseling but lower intensity services than psychotherapy. In recent years, awareness of this type of intervention has increased in Switzerland due to changes in legislation (Swiss Criminal Code art. 55a) and the provisions of the Istanbul Convention. The possibility of ordering attendance at this type of intervention is now specifically mentioned in several legal titles of the Swiss Criminal Code. Furthermore, the website of the Office of Corrections and Rehabilitation of the Canton of Zurich offers various information materials on the referral process, including inclusion and exclusion criteria for program participation.

Next, case managers determine the eligibility of individuals for the intervention based on case files and interviews. A risk assessment is conducted during the screening process following a structured professional judgement approach. Exclusion criteria for participation in the intervention are: (a) insufficient German language skills, (b) denial of intimate relationship conflicts, (c) suffering from severe mental disorders (e.g., psychotic, affective, or other severe disorders), (d) participation in psychotherapy that is also intended to reduce recidivism (except for addiction treatment, which may be combined with the intervention program), (e) an acute risk of committing serious offenses (e.g., homicide, aggravated assault, and rape), (f) having an exclusive or distinct intervention need for sexual offending, and (g) having an exclusive intervention need for environmental risk factors for recidivism (e.g., work, finances, or housing). In exceptional cases, the intervention program is delivered to individuals with insufficient German language skills in the presence of a professional interpreter. Importantly, participation in the intervention does not require participants to have confessed to the alleged IPV offenses, to be aware of the problem, or to be considering changing behavior. Based on the eligibility assessment, the case managers sent an evaluation report to the referring authority, documenting their decision regarding program participation and recommending additional or other measures to prevent IPV recidivism if suitable. Eligible individuals are required to participate in the program, and sessions are rescheduled when participants fail to attend.

The intervention program consists of an introductory phase and five training phases. In the introductory phase, participants are provided with information about domestic violence, relevant legislation, and the intervention program. The training phases involve:

1. *Understanding problematic behavioral patterns:* Participants identify their own risk factors for IPV recidivism, their motives for violence, their behavior in high-risk situations, and the consequences of their problematic behavior.
2. *Setting goals:* Participants analyze the costs and benefits of their problematic behavior and set goals toward change.
3. *Developing strategies to reduce the risk for IPV recidivism:* Participants learn a variety of skills to prevent and manage high-risk situations, such as emotion regulation, effective communication, and conflict resolution.
4. *Specifying relapse prevention plans:* Participants specify relapse prevention plans that help them recognize and manage high-risk situations.
5. *Maintaining change and reviewing the relapse prevention plans:* Participants check in with their intervention providers during follow-up to review the skills learned during the program and to discuss their relapse prevention plans.

A final report containing information on the intervention progress and outcome in terms of changes on risk factors for IPV recidivism is sent to the referring authority once the intervention and follow-up period have been completed. If participants do not comply with the intervention regimen, the referring authority is informed immediately. Please contact the corresponding author for further information on the program curriculum.

***Item 5: Intervention Providers***

The intervention is delivered by human service professionals who have a bachelor’s or master’s degree in social work, social pedagogy, or psychology and basic knowledge and skills in forensics. Intervention providers are employed by the Probation and Corrections Services and conduct regular case consultations with colleagues. The competence of professionals to deliver the intervention was established through education and training but was not measured in this study. To date, new intervention providers also complete at least six days of specialized training followed by six supervision sessions from qualified personal.

***Item 6: Delivery Modes***

The intervention is delivered in individual or group settings with up to 10 participants. Individual sessions are conducted one-to-one, whereas group sessions are led by two male or one male and one female co‑facilitator.

***Item 7: Locations and Funding of the Intervention***

The intervention is delivered in the community at the headquarters of the Office of Corrections and Rehabilitation of the Canton of Zurich in Switzerland. In exceptional cases, the intervention may be delivered in correctional institutions. If the public prosecutor’s office or court has ordered the defendant to attend the intervention in the summary penalty order or in the verdict, the program is only partially publicly funded and participants have to bear part of the costs. In all other cases, the intervention is fully financed by the cantonal authorities.

***Item 8: Intervention Intensity***

In the group setting, the intervention consists of 16 weekly sessions of 2.5 hours each and three follow-up sessions of one hour each, which are typically conducted at three‑month intervals. The follow-up sessions may be conducted at shorter intervals if there is less time to intervene due to the referral status. In the individual setting, the intervention consists of 15 to 25 sessions of 1-hour each. Follow-up sessions are always conducted in the individual setting. The average duration of the intervention is 1-year.

***Item 9: Tailoring of the Intervention***

With regards to program curriculum, all participants receive the same intervention. However, the delivery mode is tailored to participants. The intervention is usually delivered in groups, unless individuals are not suitable for this setting. The intervention is delivered in the individual setting, if participants have insufficient German language skills, have perpetrated violence within same-sex relationships, are diagnosed with social anxiety disorder, or are at higher risk of IPV recidivism. Higher-risk participants attend the program individually in order for intervention providers to deliver more intense and broader services. Furthermore, group sessions are supplemented by individual sessions when participants fail to attend sessions, when individuals with poor German language skills require additional sessions to process the intervention content, when other needs that negatively impact participation in the program should be targeted (e.g., child custody proceedings), or when there are sensitive topics that cannot be addressed in the group setting. The individual setting allows intervention providers to delve into the topics most relevant to the participant, while skimming over less relevant topics.

***Item 10: Modifications During the Study Period***

There were no major changes to the intervention during the study period. However, the workbook was revised in 2013. Modifications included changes to the wording and graphic design of the worksheets as well as use of another personality assessment and classification of character traits. Apart from this, there were no changes to the content of the workbook.

***Item 11: Program Adherence and Integrity (Planned)***

With regard to intervention providers, strategies used to maximize program integrity include their education and training and the use of a manual. With regard to participants, program integrity is maximized by rescheduling sessions when participants fail to attend and by distributing workbooks, including homework assignments to be completed during the course of the intervention.

Adherence to the intervention during the study was assessed by documenting the intervention duration and whether participants started the program, completed a clinically meaningful number of sessions, or completed all sessions. Reasons for non-adherence to the intervention were not consistently documented.

***Item 12: Program Adherence and Integrity (Actual)***

Participants’ adherence to the intervention is reported in the body of the paper. In the majority of cases, the reasons for non-adherence were unknown. However, the documented reasons include that participation in the program was ultimately not imposed or revoked by the referring authority, that another type of preventive intervention was ordered, that the participant’s place of residence changed, and that participants had died.

**Appendix B**

**Flow of Participants Through the Quasi-Experiment**


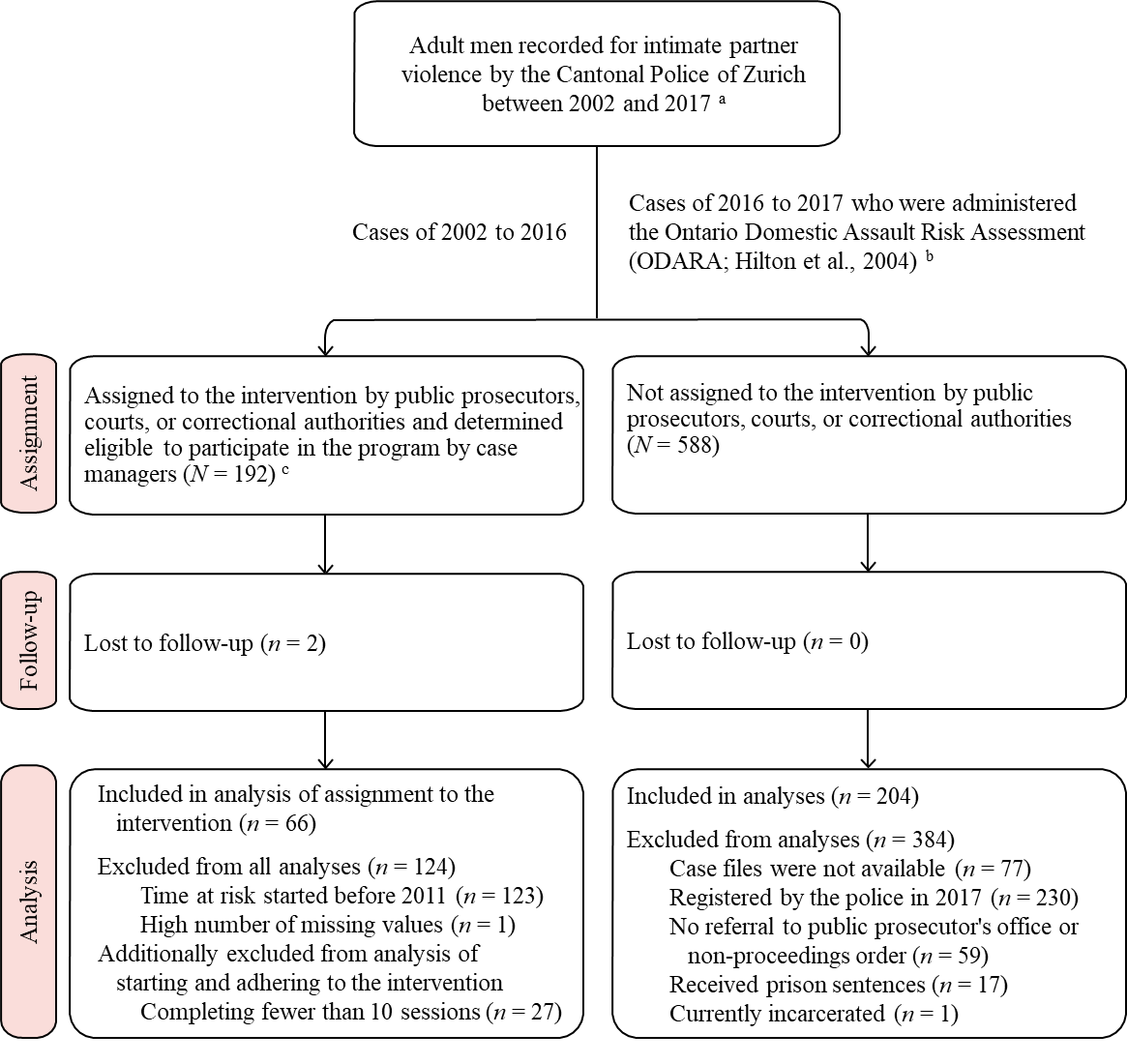


*Note.* This flowchart was adapted from the Journal Article Reporting Standards for Quantitative Research (JARS-Quant; Appelbaum et al., 2018), an adaptation of the Consolidated Standards of Reporting Trials (CONSORT) 2010 statement flowchart (Schulz et al., 2010).

^a^ Please note that the two study groups are not fully comparable. In contrast to the control group, individuals in the intervention group may also have been recorded for intimate partner violence by another police force because any correctional authority in Switzerland may refer individuals to the intervention. ^b^ Similarly, not all individuals in the intervention group have perpetrated an index offense involving physical contact or a threat of death with a weapon. ^c^ While the control group includes both individuals who are eligible and ineligible to participate in the intervention, the intervention group only includes eligible individuals.

**Appendix C**

**Results and Discussion of Sensitivity Analyses**

The results for the unimputed data are reported in the main body of the paper, while the results for the imputed data are reported in Table C1. Group membership was statistically significant in the multivariable models on the unimputed data for both group comparisons. In the multivariable models on the imputed data, group membership remained statistically significant for the comparison between the intervention assignment and control group (*F*(4, 270) = 3.88, *p* = .004). However, group membership was only marginally significant for the comparison between the intervention completion and control group (*F*(4, 243) = 2.82, *p* = .024). Furthermore, none of the other covariates were statistically significant in the multivariable models on the unimputed data. However, the nationality of perpetrators reached statistical significance in the multivariable models on the imputed data for both group comparisons.

Overall, the results in the statistical model with and without missing data are similar. In both multivariable models for the comparison of the intervention completion and control group, group membership was at the threshold of statistical significance, crossing it in one model and not crossing it in the other. In addition, the nationality of perpetrators was already marginally significant in the multivariable models on the unimputed data.

The results of the sensitivity analyses provide some indication that the IPV recidivism rate at 2-year follow-up was lower for Swiss nationals than for foreign nationals. Given the small sample size, future research should explore whether this trend reflects true differences in IPV recidivism. One explanation for this finding may be that foreign nationals are at increased risk of IPV recidivism and therefore require a higher intensity of services than Swiss nationals to achieve comparable outcomes. This explanation is consistent with the risk principle (Andrews & Bonta, 1990; Bonta & Andrews, 2023) and with previous research showing that immigration may increase the risk of perpetrating IPV and that many immigrants have a broad range and high prevalence of risk factors, some of which are specific to this subgroup (Ayubi & Satyen, 2024). Another explanation may be that the criminal justice response to IPV is less effective in reducing IPV recidivism among foreign nationals than among Swiss nationals. The literature suggests that effective IPV interventions should not only adhere to the risk principle, but also to the need and responsivity principles (Andrews & Bonta, 1990; Bonta & Andrews, 2023; Travers et al., 2021). To achieve comparable outcomes between foreign nationals and Swiss nationals, it may be important to ensure that services are appropriately adapted to an individual’s racial or ethnic identity, culture, and immigration status (Emezue et al., 2021; Turhan, 2020).

Since the effectiveness evaluation was completed, the intervention has been refined. Refinements have been made not only to the content of the program but also to the languages available. In particular, the Government Council of the Canton of Zurich decided that the intervention program should be offered in the most commonly spoken languages of the target population (Gewalt gegen Frauen, Umsetzung der Istanbul-Konvention im Kanton Zürich, Massnahmen und Stellenplan, 2021). In the past, the intervention program was only available in German and facilitators had to work with a professional interpreter to deliver the intervention or participants had to speak German to be eligible to participate in the program. As such, the intervention program was not accessible to everyone in the target population and non-native speakers attending the program may not have fully benefitted from the intervention. As Bonta and Andrews (2023) note, culture is one of the specific responsivity factors to which correctional services should be adapted in order to be effective, and past researchers have suggested that providing culturally sensitive services involves overcoming language barriers to participation in IPV interventions (Emezue et al., 2021; Turhan, 2020). Given the trend towards higher IPV recidivism among foreign nationals compared to Swiss nationals in this study, tailoring the intervention to the nationality of participants by offering the program in different languages seems a promising avenue for improving the effectiveness of the intervention program.

**Table C1**

*Results of the Multivariable Cox Proportional Hazards Regression Analyses on the Imputed Data for Both Group Comparisons*

| Variable | HR | *SE* | *z* | *p* | 95% CI |
| --- | --- | --- | --- | --- | --- |
| Comparison intervention assignment vs. control group | | | | | |
| Group membership | 0.23 | 0.12 | -2.81 | .005 | [0.08; 0.64] |
| Perpetrator age ^a^ | 0.47 | 0.24 | -1.48 | .139 | [0.17; 1.28] |
| Perpetrator Swiss nationality | 0.51 | 0.16 | -2.10 | .036 | [0.27; 0.96] |
| Prior convictions for violent or  sexual offenses | 1.52 | 0.58 | 1.11 | .266 | [0.72; 3.20] |
| Comparison intervention completion vs. control group | | | | | |
| Group membership | 0.31 | 0.19 | -1.94 | .052 | [0.10; 1.01] |
| Perpetrator age ^a^ | 0.49 | 0.25 | -1.38 | .166 | [0.18; 1.34] |
| Perpetrator Swiss nationality | 0.51 | 0.17 | -2.07 | .038 | [0.27; 0.96] |
| Prior convictions for violent or  sexual offenses | 1.44 | 0.56 | 0.92 | .358 | [0.66; 3.11] |

*Note.* HR = hazard ratio; CI = confidence interval.

^a^ Perpetrator age was not normally distributed and thus, log transformed.

**Appendix D
Equations for the Cost-Benefit Ratio**

A comprehensive cost analysis estimated the tangible costs for IPV in Switzerland at CHF 164 to 287 million per year and the intangible (lifetime) costs at CHF 1,969 billion (Fliedner et al., 2013). In this study, the tangible costs included direct costs for justice, healthcare, and support services as well as indirect costs for lost productivity for the reference year of 2011, while the intangible costs monetized lost quality of life for victims within that year. In 2011, the police recorded 12,123 IPV offenses (Federal Statistical Office, 2024c). Given this number, the estimated average tangible and intangible costs per offense amount to CHF 13,528 to 23,674 and CHF 162,419, respectively. By contrast, the personnel costs for delivering the intervention program range from CHF 3,200 per person in the group setting to CHF 4,100 per person in the individual setting. Considering the results of the present study showing that 6 individuals need to be assigned to prevent one re-offense for IPV, a preliminary estimation of the costs suggests that the intervention program achieves an estimated positive cost-benefit ratio of 1 to 7. This rough estimate considers the intangible costs for IPV and suggests that for every Swiss franc invested, seven Swiss francs are returned.

Number needed to assign:

- 46 (individuals in the control group who recidivated) : 204 (total number of individuals in the control group) = 0.2255 (risk in the control group)
- 4 (individuals in the intervention group who recidivated) : 66 (total number of individuals in the intervention group) = 0.0606 (risk in the intervention group)
- 0.2255 (risk in the control group) - 0.0606 (risk in the intervention group) = 0.1649 (risk difference)
- 1 : 0.1649 (risk difference)  = 6.06 (number needed to assign)

Costs for IPV:

- 164,000,000 (lower bound tangible costs) : 12,123 (number of IPV offenses) = 13,528 (average tangible costs per offense)
- 287,000,000 (upper bound tangible costs) : 12,123 (number of IPV offenses) = 23,674 (average tangible costs per offense)
- 1.969,000,000 (intangible costs) : 12,123 (number of IPV offenses) = 162,419 (average intangible costs per offense)
- 16.50 (number of re-offenses prevented per 100 participants assigned) x 162,419 (average intangible costs per offense) = 2,679,914 (intangible costs for 16.50 re-offenses)

Costs for the intervention program:

- 3,200 (costs per person in group setting) x 75 (individuals participating in group setting per 100 participants) + 4,100 (costs per person in individual setting) x 25 (individuals participating in individual setting per 100 participants) = 342,500 (costs of the intervention per 100 participants)

Cost-benefit ratio:

- 2,679,914 (intangible costs for 16.50 re-offenses) : 342,500 (costs of the intervention per 100 participants) = 1:7.82 (cost-benefit ratio)
